# *In vitro* Inhibition of Border Disease Virus Replication With Lentivirus-Mediated shRNAs

**DOI:** 10.3389/fvets.2021.708591

**Published:** 2021-08-10

**Authors:** Mohammad Javad Hajihasani Arani, Azam Mokhtari, Behnaz Saffar, Leila Asadi Samani

**Affiliations:** ^1^Department of Genetics, Faculty of Science, Shahrekord University, Shahrekord, Iran; ^2^Department of Pathobiology, Faculty of Veterinary Medicine, Shahrekord University, Shahrekord, Iran; ^3^Zoonotic Disease Research Institute, Shahrekord University, Shahrekord, Iran; ^4^Biotechnology Research Institute, Shahrekord University, Shahrekord, Iran

**Keywords:** BDV, lentiviral plasmid, NS3, RNAi, TCID50, real-time PCR

## Abstract

**Background:** Border disease is believed to be one of the most important diseases in the animal husbandry industry, which has not yet been eradicated in Iran. The development of approaches based on the application of interfering RNA (RNAi) for antiviral therapy has attracted a great deal of attention over the recent years. The present research was conducted to design, construct, and apply shRNA against the NS3 gene of BDV to evaluate the prevention of BDV proliferation in the cell culture system. For this purpose, the suitable oligonucleotide sequence of NS3 gene coding was selected utilizing BDV- X818 strain. Afterwards, using shRNA design software, shRNA molecules were designed and synthesized. These shRNAs were cloned into the desired vectors and were finally transfected in HEK293T cells employing the third generation of lentiviral packaging system. Subsequently, these shRNA expressing lentiviruses were transduced to the MDBK cell line to challenge to border virus. In order to evaluate the efficacy of shRNAs, the viral infectious titer and RNA copy number were calculated with TCID50 and Real-time RT-PCR tests, respectively.

**Results:** The results revealed that shRNAs 1, 2, and 3 decreased viral RNA by more than 90% compared to the control groups. BDV titer noticeably decreased after the challenge with shRNAs 1, 2, and 3 from ~88% up to 99% in comparison with the control groups.

**Conclusions:** Overall, it could be concluded that RNAi may be considered as a strong treatment proposal against viruses, such as BDV.

## Introduction

Border disease is a viral sickness of small ruminants. Infertile ewes, abortion, stillbirth, and the birth of tiny, faint lambs that may have a vibration, unusual body conformation, and hairy fleeces are the most prevalent manifestations of the disease. Border disease virus (BDV), such as other pestiviruses, may be either cytopathogenic (CP) or non-cytopathogenic (NCP) and has seven clusters (BDV 1–7) (1). BDV could be transferred among cattle and both sheep and goats (2, 3). The disease is prevalent all over the world and leads to basic economic losses in animal farms due to certain complications, such as weak reproduction in herds and expensive follow-up tests (4). BDV is a member of the genus pestivirus from the family of Flaviviridae (1). The viral genome is ssRNA+, which encodes one polyprotein that is cleaved into 11 proteins after translation (5′- Npro, C, Erns, E1, E2, p7, NS2-3, NS4A, NS4B, NS5A, and NS5B-3′) (2).

NS3, NS4A, NS4B, NS5A, and NS5B are conserved and necessary during RNA replication (5). In CP BDV, NS3 is expressed, while NS2-3 expression is established in both NCP BDV and CP BDV (6). NS2-3 cleavage is very important for BDV replication since free NS3 is an obligatory replicase whose performance cannot be replaced by NS2-3 (7).

The pestiviral NS3 acts as serine protease, RNA helicase, and nucleoside triphosphatase (NTPase). Its N-terminal is a protease for the cleavage of BDV polyprotein. The helicase and NTPase activities belong to the C-terminal of the NS3 protein. The C-terminal domain of NS3 has a vital role for BDV replication due to RNA helicase activity (8).

The RNAi pathway is a process in which two-strand RNA molecules, with specific length and secondary structure, degrade the homologous RNA targets or suppress the expression of their complementary genes. This pathway in the cell naturally aims to protect the genome against external genetic threats, such as viral genes and transgenes, and internal threats, like transposons. Furthermore, it is responsible for the regulation of gene expression and development (9).

Nowadays, the RNA interference pathway has become a mere biological phenomenon as a powerful therapeutic tool for treating a wide range of diseases, including viral infections. Ease of use, rapidity, excellent performance, and remarkable specificity once exerted at various phases of virus–host interplay are some of the potential advantages of the RNAi pathway as an antiviral approach over traditional methods, for instance, antiviral drugs or vaccines (10).

RNAi is supplied to the cell from short interfering RNA (siRNA) or short hairpin RNA (shRNA) to downregulate the expression of target genes. Several studies have shown that shRNA offers advantages in silencing the target genes, such as stability, cost-effectiveness, and simplicity of delivery. The shRNA expression cassettes are consistently integrated into the host cell genome and suppress the expression of the target gene by homologous mRNA degradation without any changes in other mRNAs. Thus, RNAi seems to be an appropriate option as a therapeutic method for protecting plant and animal species against various viruses (11, 12).

In the present study, having designed shRNAs against the BDV-X818 NS3 gene, we generated lentivirus-expressing shRNAs in HEK 293 T cells in order to inhibit BDV multiplication. Finally, we evaluated the rate of BDV replication in MDBK cells.

### Design and Preparation of BDV-NS3 shRNAs

In order to design the desired shRNA against border disease, increase the stability of shRNA molecules, reduce off-target effects in target cells, and improve shRNA function as described by Tom Tuschl (13), Mcintyre et al. (14), and Taxman et al. (15), the following software and web databases were utilized:

- NCBI database at gov.nih.nlm.ncbi.www to obtain the BDV-NS3 gene sequence and align it with the host genome in order to eliminate shRNAs having much homology with sheep genomic and transcripts
- WI siRNA Selection Program (www.rnaidesigner.thermofisher.com/rnaiexpress), BLOCK-iT™ (www.sirna.wi.mit.edu), RNAi Web Designer, and siRNA Wizard (www.invivogen.com/sirnawizard) for shRNAs design
- Clustal omega web software-3 (http://www.ebi.ac.uk/Tools/msa/clustalo) to study the conserved areas of NS3 gene
- Workbench Genomics CLC software to predict the second structure of border virus NS3 gene

shRNAs were evaluated after being designed with the BLAST tool at the NCBI database until shRNAs <15 nucleotides shared with sheep genome mRNAs were selected (16).

### Preparation of Lentiviral Plasmids Expressing shRNAs and BDV-NS3

Based on the BDV-X818-NS3 gene (accession number AF037405.1), three shRNA candidate sequences were selected, synthesized, and cloned into pCDH-CMV-MCS-EF1-cGFP-T2A-Puro lentiviral plasmid (provided by Bonbiotech) digested with EcoRI and BamHI. On the other hand, the domain of the HELICc binding site of BDV-NS3 was synthesized and cloned in this plasmid in the same way. This vector possesses the CopGFP gene which is controlled with an EF1 promoter for monitoring of the shRNA transfection efficiency. It also has puromycin resistance cassette.

**Figure 1:**
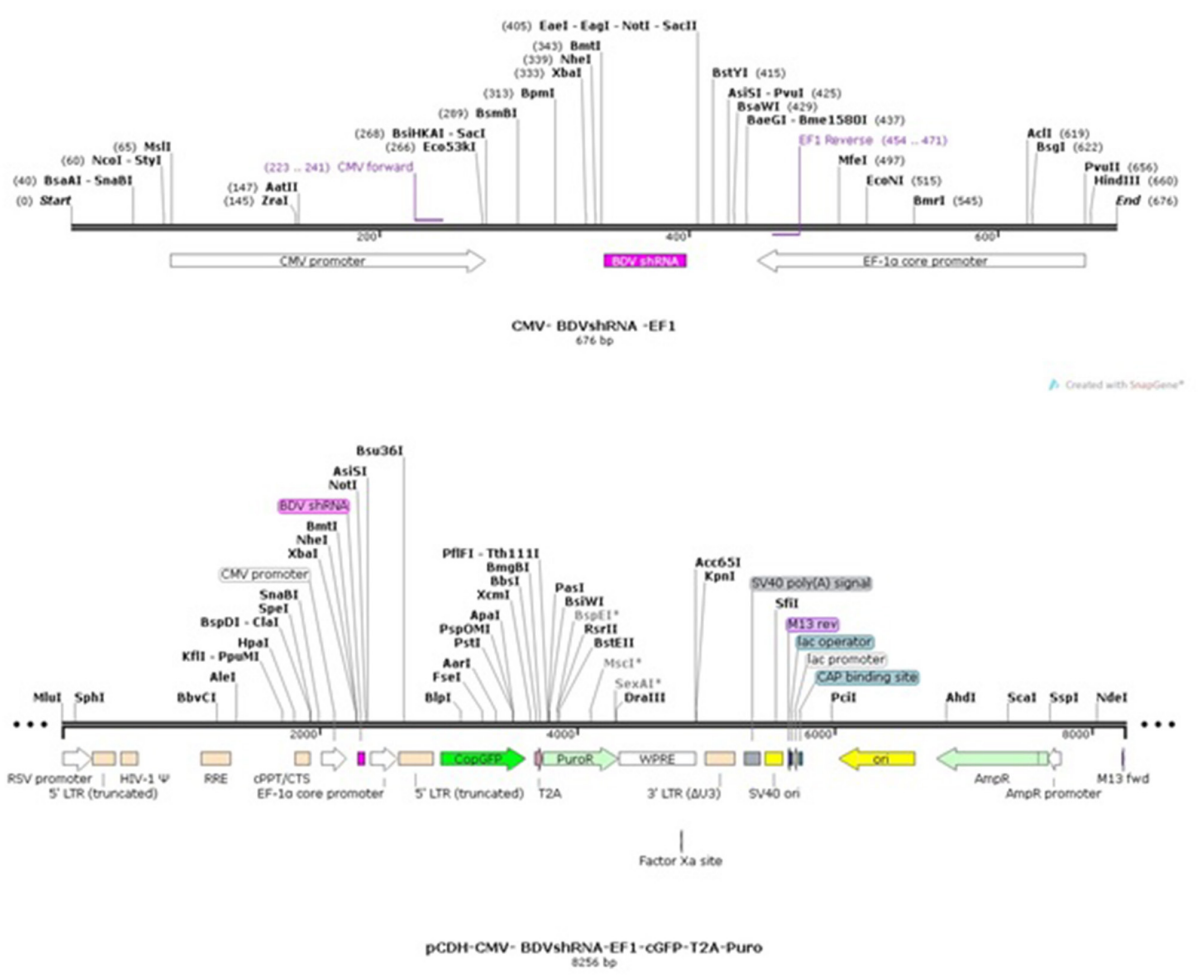
pCDH-CMV-shRNA-EF1-cGFP-T2A-Puro map.

Following the cloning, the prepared recombinant plasmids were transformed in the DH5α strain of *Escherichia coli*, and the cloning accuracy was confirmed by sequencing. These lentiviral transfer plasmids were then applied for transfection in the third generation of the lentiviral packing system. As a mock, pEZX-MR03 was implicated, which contains the EGFP gene controlled with a CMV promoter lacking any significant shRNA sequences (Figure 1) (17).

### Cell and Virus Propagation

The BDV-X818 standard strain was obtained from Animal Health and Veterinary Laboratories Agency. The titer of the virus stock was 4 × 10^2.5^ TCID50/ml. BDV-X818 was used at a multiplicity of infection (MOI) of 5 for the *in vitro* challenge with shRNAs (18). HEK 293 T cells (ATCC number: CRL-1573) were applied for the preparation of lentiviral vectors, and MDBK cells (ATCC number: CCL-22) were employed for the inoculation of BDV, titration, and *in vitro* challenge with shRNA-expressing lentivectors.

### Production of Lentivectors

In the present study, the third generation of the lentiviral packaging system was used to prepare the lentivirus-expressing anti-BDV-NS3 shRNAs and BDV-NS3 HELICc and binding site domain.

Primarily, pCDH transfers lentiviral plasmids expressing anti-BDV-NS3 shRNAs, and BDV-NS3 HELICc was prepared by cloning; the verification of cloning proses was performed by sequencing.

Afterwards, the HEK 293 T cells were cultured in 10-cm plates and in a confluency of approximately 70–80%; they were co-transfected by three lentiviral plasmids with the names psPAX—a packaging vector (21 μg), pMD2.G—a pseudo-type (VSV-G) envelope vector (10.5 μg), and pCDH containing anti-BDV-NS3 shRNAs or BDV-NS3 HELICc and binding site cassette (or pEZX-MR03 as a mock) as transfer vector (21 μg) using the calcium phosphate protocol according to the instructions of Bonbiotech company (Iran).

At 48 and 72 h after co-transfection, the GFP expression was evaluated with fluorescent microscopy, and if it was sufficient, at the same time, the supernatants were collected and centrifuged (1,000 *g*, 15 min). They were then kept at −70°C until the challenge by BDV (19).

### Production of Cells Expressing BDV-NS3 HELICc and Binding Site Infection With shRNA

For induction of BDV-NS3 HELICc and binding site expression in MDBK cells, the lentiviral vectors expressing it (MOI = 1) were inoculated in trypsinized MDBK cells (3 × 10^5^ cells per well of a six-well plate). The cells were cultured in 1 ml of Dulbecco's modified Eagle's medium (DMEM; Gibco, America, catalog no. 116–12800) and supplemented with 10% fetal bovine serum (FBS). At 12 h post-infection (pi), 2 ml of fresh DMEM replaced the previous medium. At 48 and 72 h pi, fluorescent microscopy of the cells was performed (20). Following RNA extraction from the infected cells using the RNeasy mini kit (Qiagen, Crawley, UK) and DNase (Qiagen, Crawley, UK) treatment, RT-PCR was performed for detection of infection by employing the following primers: F: 5′-GGGACCGAGACAGTCAACTT-3′ and R: 5′-GGTCCCGTTGTTGTTGTTGA-3′ for each sample and positive (BDV-infected MDBK cells) and negative (MDBK cells without any infection) controls. The PCR thermal cycling included denaturation for 2 min at 95°C followed by 30 cycles at 95°C for 30 s, 53°C for 30 s, and 72°C for 30 s, followed by a final extension at 72°C for 5 min.

Subsequently, MDBK cells expressed BDV-NS3 HELICc, and binding sites (3 × 10^5^ cells in each well of a six-well plate, 90% confluency) were infected by shRNA-expressing lentiviral vectors (MOI = 5, three replicates per vector) cultured in 1 ml of DMEM supplemented with 3% FBS and incubated at 37°C with 5% CO_2_. The medium was changed at 12 h pi with the same medium + 10% FBS and 1X penicillin streptomycin (Sigma, America catalog no. 116-12800). At 12 h after changing the medium, the cells were re-infected with the same conditions (21).

### BDV Challenge With Lentivirus-Expressing shRNAs

A culture of 3 × 10^5^ MDBK cells per six-well plate in DMEM + 10% FBS + 2 mM L-glutamine + 1X Pen-Strep and 2.5 mg/L amphotericin B was prepared and incubated at 37°C with 5% CO_2_ for 24 h (22). Afterwards, the medium was changed, and infection with shRNA-expressing lentiviruses was carried out (three replicates per shRNA). At 24 h following the first lentiviral inoculation, the infection was repeated.

GFP expression in MDBK cells was evaluated by observation by means of a fluorescent microscope at 48 h pi. Once GFP expression was sufficient, the challenge with BDV was done as described previously. At 72 h after the challenge, the cellular morphology and the development of cytopathic effects (CPEs) were evaluated. The positive (MDBK cells infected with only BDV) and negative (MDBK cells with scrambled vector infection or without any viral infection) controls were included in the assay (21).

### BDV Titration With TCID50 Method

To investigate the effect of shRNAs on CPE development and variation in BDV titer, TCID50 assay was carried out in 96-well plates. Three replicates were considered per dilution and also per infection condition. Ultimately, the Spearman–Karber formula was applied for viral titer calculation (23).

### RT-qPCR

At 72 h following the challenge, total RNA was extracted from MDBK cells by means of RNeasy mini kit (Qiagen, Crawley, UK), and DNase treatment (Qiagen, Crawley, UK) was performed. Subsequently, reverse transcription reaction was performed, utilizing 1 μg total RNA, Superscript II (Invitrogen), and oligo (dT) for 1 h at 42°C. For the RT-qPCR assay, Power SYBR Green Mastermix (Applied Biosystems, Warrington, UK) was applied. The sequences of the primers were designed using the GenScript real-time RT-PCR Primer Design web tool. They were as follows: forward: 5′-ATTCGTGCCCACCAGGAATA-3′, reverse: 5′-CAAGTTAGCCGGGTCCTCTC-3′, *Bos taurus* GAPDH forward: 5′- TGAGGACCAGGTTGTCTCCT-3′ and *Bos taurus* GAPDH reverse: 5′-CACCCTGTTGCTGTAGCCAAAT-3′. The reactions occurred on an iQ5 real-time RT-PCR detection system and were done in triplicate. The temperature conditions performed by the device were as follows: 30 s denaturation at 95°C followed by 40 cycles of 95°C for 30 s, 53°C for 45 s, and 72°C for 1 min. Gene multiplication was determined by melting the curve profile. Cycle threshold (Ct) values were normalized to GAPDH, and a relative BDV RNA level was calculated based on the ΔΔCt method (23). The ratio of fold-change values of test to control was calculated. Multiplying the ratios by 100, a percentage reduction in BDV replication was then obtained.

## Results

### Reduction of BDV NS3 Expression by shRNA

**Figure 2:**
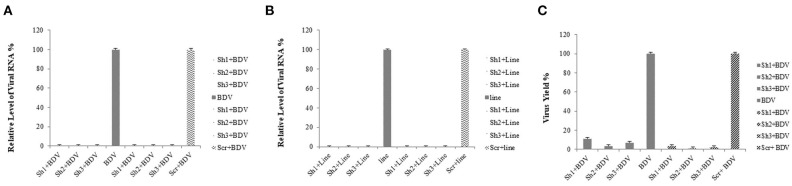
**(A)** GFP expression after the co-transfection of HEK293T cells: GFP expression by transfected cells with psPAX and pMD2.G as packaging vectors and pCDH carrying BDV-ShRNA1, pCDH carrying BDV-ShRNA2, and pCDH carrying BDV-ShRNA3 and pEZX-MR03 (as a mock), respectively. **(B)** GFP expression after titration of HEK293T cells with lentivectors: GFP expression by infected HEK293T cells with lentivector expressing BDV-ShRNA1, BDV-ShRNA2, and BDV-ShRNA 3 and scrambled lentivector, respectively. **(C)** The same pictures taken with a light microscope.

At 72 h after the co-transfection of lentiviral plasmids, including transfer plasmids carrying BDV-ShRNA1, BDV-ShRNA2, BDV-ShRNA 3, and mock in HEK293T cells, the EGFP expression indicated that the lentiviral vectors were successfully produced. At 72 h following the infection of MDBK cells with the prepared lentiviruses, the GFP expression indicated that the lentiviral vectors were efficiently integrated into the cells. At 72 h after the infection, the cell viability of MDBK cells co-infected with BDV-shRNAs and BDV was more in comparison to that of the cells co-infected with the scrambled lentivector and BDV or BDV alone. Furthermore, the effects associated with the development of BDV cytopathic were less in the BDV-infected cells expressing shRNAs (Figure 2).

**Figure 3:**
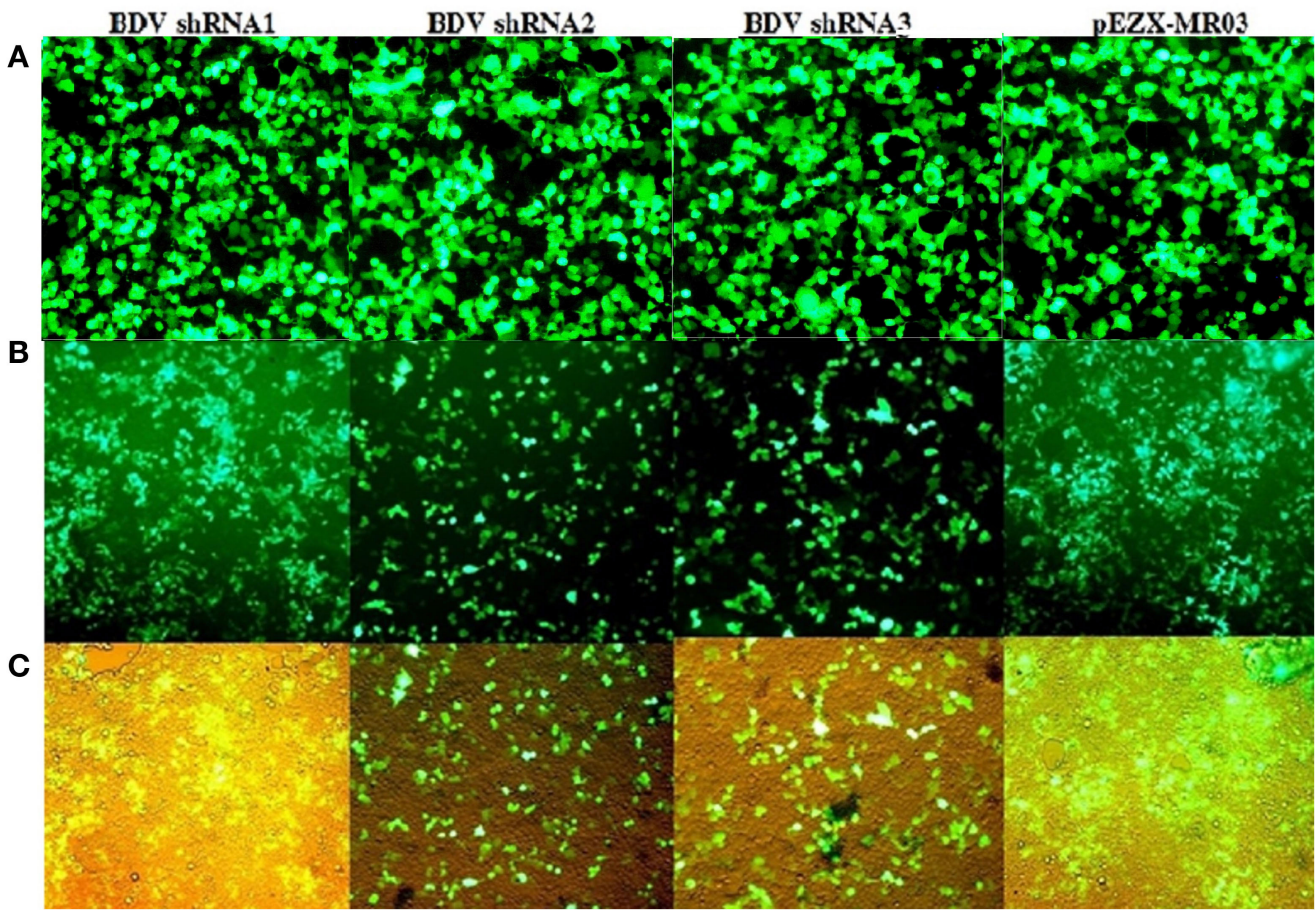
**(A)** GFP expression after the infection of MDBK cells with lentivectors: GFP expression by infected MDBK cells with lentivector expressing BDV-ShRNA1, BDV-ShRNA2, and BDV-ShRNA 3 and scrambled lentivector, respectively. **(B)** The same pictures taken with a light microscope. **(C)** Lentiviral ShRNA-GFP expression of uninfected MDBK cells: GFP expression by uninfected MDBK cells with lentivector expressing BDV-ShRNA1, BDV-ShRNA2, and BDV-ShRNA 3 and scrambled lentivector, respectively.

The results of the real-time RT-PCR confirmed the efficiency of lentiviral-expressed shRNAs on BDV gene knockdown. Using real-time RT-PCR, we observed a reduction in the expression of viral RNA in the cells treated with shRNAs 1, 2, and 3 (93.76, 96.64, and 97.75%, respectively) in comparison with that in the cells infected with BDV and 90.89, 95.10, and 96.72% compared to that in the cells infected with BDV and scrambled vector, respectively (*P* < 0.05). Additionally, after the calculation of BDV infectious titer using the TCID50 method, it was observed that the BDV titer remarkably decreased after the challenge with shRNAs 1, 2, and 3 (88.87, 96.48, and 92.96%, respectively, in comparison with the cells infected with BDV and 96.49, 98.89, and 97.78% compared to the cells infected with BDV and scrambled vector, respectively). The results of CPE indicated a reduction in virus yields in each group (Figure 3).

### Reduction of BDV-NS3 HELICc and Binding Site Expression by shRNA

**Figure 4:**
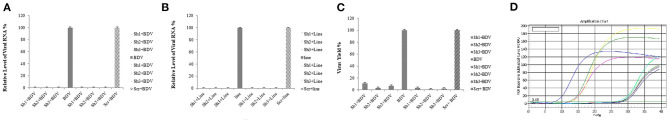
Reduction of viral RNA and titer by lentiviral-expressing shRNA in MDBK cells. The total RNA was extracted, and real-time RT-PCR was performed for the determination of relative RNA. The amplification chart is shown in **(D)**. All the values are displayed in percentages of controls (cells infected with BDV and those infected by the scrambled vector). shRNAs 1, 2, and 3 markedly reduced the expression of the selected gene and viral titers compared to the control groups compared to the cells without any infection and those infected by the scrambled vector **(A,C)**. The same reductions in viral RNA expression was observed in MDBK cells expressing BDV-NS3 HELICc and the binding site after applying shRNAs 1, 2, and 3 **(B)**.

Real-time RT-PCR was performed to detect possible differences in the transcription of the mentioned domain after the preparation of MDBK cells constantly expressing BDV-NS3 HELICc and binding site by lentiviral vectors and the transduction of these cells and controls (MDBK cells infected with BDV and those infected with mock vector) with lentiviral-mediated shRNAs. Moreover, we demonstrated that shRNAs 1, 2, and 3 noticeably decreased the gene expression. The reduction rate of the selected BDV NS3 domain with shRNAs 1, 2, and 3 was approximately 99% for the three shRNAs compared to both control groups (*P* < 0.05) (Figure 4).

## Discussion

*Pestivirus* infections cause a huge economic impact on livestock production. Despite the fact that there are vaccines for bovine viral diarrhea virus (BVDV), the demand for anti-BDV vaccines is limited, and those produced are inactivated products. There are no commercial live attenuated or recombinant subunit vaccines for BDV. The BVDV vaccine for cows is not recommended for use in sheep since border disease viruses, which are usually common in sheep population, are genetically different from the most common strains of BVDV (3, 24, 25). On the other hand, it is important to note that no systematic antiviral drugs have been reported for border disease; however, since BDV has a very close relationship with its family members, BVDV and CSFV, the drugs used for them could be implicated for BDV. These compounds target cellular proteins which play roles in viral maturation or viral encoded enzymes, such as the NS3 or the NS5B RNA-dependent RNA polymerase (RdRp)—for instance, DB772 and BPIP are two chemical compounds introduced as antiviral agents for BDV (26). However, it must be considered that the unwanted side effects may be due to the use of these antiviral compounds (27). Treatments based on altering the expression of the target genes and employing the natural intracellular pathway have fewer adverse effects than other therapies. Therefore, over the recent years, RNAi has been considered an effective treatment strategy (27). Accordingly, in the present study, this strategy was chosen to suppress BDV replication. Despite the advantages of the therapeutic application of siRNA molecules against acute viral infections, the use of shRNA molecules, which triggers a more stable gene expression and less off-target effects, seems to be more appropriate. On the other hand, to date, certain features of lentiviral vectors, including extensive tissue tropism, persistent gene expression, and low carcinogenic risk, have been reported in the *in vivo* conditions (28). Considering the advantages of lentivectors and shRNA molecules, in the present study, lentivector-based shRNAs were selected to suppress viral gene expression.

In the studies based on gene therapy against pestiviruses, owing to the conservation of the non-structural proteins and their functional roles in the virus life cycle, they have been selected as the targets. In the present study, given the vital roles of NS3 in pestiviral pathogenicity, it was selected to be downregulated by lentivirus-mediated shRNAs.

Unfortunately, we did not find any reports on the application of interfering RNAs against border disease virus. However, there are a number of research involving the application of RNAi against the members of Flaviviridae—for example, the inhibition of BVDV-1 replication in the cell culture system has been published using siRNAs targeting the 5′-UTR, capsid (C), NS4B, and NS5A regions by Lambeth et al. (29). They applied a specific analysis approach of RNAi efficiency by developing a cell line expressing BVDV sub-genomic replicons (29). In another work, Mishra et al. determined the inhibitory effect of siRNAs targeting BVDV-Env and the 5′-UTR in the cells expressing subgenomic replicons and cells infected by BVDV (30). One of the methods for assessing the effectiveness of induced RNAi is their evaluation in cell lines expressing the target genes. Hence, in the present study, such cell clone was prepared. In the BDV-NS3 sequence, there are multiple conserved domains, among which the HELICc and nucleotide binding site domain were chosen for preparing a monitoring cell line for evaluating anti-BDV shRNAs. There are a number of reports on generating sub-replicon expressing or reporter cell lines to assess RNAi-based therapies against the members of Flaviviridae—for instance, Mokhtari et al. generated a MDBK cell line persistently expressing BVDV-5′UTR and BVDV-NS3 *via* infection with lentiviral vectors (24). Similar assessments were performed by Basagoiti et al. (31) who developed a Western Nile virus (WNV) sub-replicon-expressing cell line. This cell line was successfully implicated to assess the chemical inhibitors of the WNV strains.

Unfortunately, we did not find any report on the application of interfering RNAs for the inhibition of border disease virus; however, there are a number studies on the other members of Flaviviridae.

Xu et al. (32) suppressed the multiplication of classical swine fever virus replication with siRNAs designed for Npro and NS5B genes. They observed a 4–12- and 467-fold decrease in viral RNA. Titer was determined with RT-PCR and the TCID50 method, respectively. This suppression persisted for 72–84 h. Kapadia et al. (33) reported that HCV replication and transcription in Huh-7 cells, which continuously expressed the HCV proteins, may be significantly prevented with RNAi. In another research, Sen et al. demonstrated that anti-HCV-NS5A siRNAs characteristically suppressed NS5A transcription and translation in a human hepatoma (HepG2) cell line (34). Li et al. (35) targeted CSFV NS3, Npro, and NS5B with single, double, and quadruple anti-CSFV siRNA expression plasmids. They observed that single or multiple siRNA expression plasmids effectively suppressed the CSFV life cycle in host cells and that the suppression was noticeably more efficient once more siRNAs were used against more than one gene of CSFV. Porntrakulpipat et al. inhibited CSFV replication with synthetic siRNA targeting the C gene (36). Ni et al. showed that the dual shRNA system is a more effective approach to reducing the BVDV titer (37).

Similar to what was mentioned concerning the application of RNAi for BDV, there are no studies on the preparation of lentivectors expressing anti-BDV interfering RNAs. However, a number of reports investigating some other members of Flaviviridae have been published—for instance, Kumar et al. used LVs to deliver shRNAs against conserved domains of the JEV and WNV env gene (38). Furthermore, Kumar et al. described that the RVG lentivirus pseudotypes were more efficient for delivery into the central nervous system cells (39). In another research, Henry et al. (40) employed lentiviral viruses to infect the Huh-7 cell line using a third-generation packaging system expressing NS3, NS5b, and IRES from the 5′UTR of HCV. They evaluated the changes in gene expression after RNAi induction using flow cytometry and real-time PCR tests. Another work performed by Li et al. (41) suggested infected PK-15 cells by replication-incompetent retroviral vectors expressing siRNAs for the downregulation of CSFV Npro and NS4A proteins. They explained that, in this cell line with continuous siRNA expression, there was a 186-fold decrease in the viral genome copy number in 72 h pi and a sufficient inhibition of virus replication lasted up to 120 h (41). In the present study, a reduction of over 90% in BDV genome copy number and 88 to 90% infectious titer was observed in 72 h pi, which persisted for at least 120 h. The implication of RNAi in the present research was limited to the cell culture system. However, according to the published data of several research groups regarding transgenic animals that express shRNAs targeting a number of viral animal pathogens, we hope that RNAi treatments will be promising for *in vivo* trials. The development of a goat and a calf that expressed shRNA targeting the prion protein, the shRNA mediated suppression of porcine reproductive and respiratory syndrome in a pig, and eventually developing transgenic mice expressing two anti-FMDV shRNA are examples of the promising application of interfering RNAs *in vivo* (10). Although the utilization of interfering RNAs in animal models and natural hosts revealed significant benefits, the necessary preparations for the market must be considered before their extensive application in the livestock industry. The results of the present study supported its subsequent application in terms of *in vivo* and field conditions. In general, the isotropy of further understanding of the mechanisms involved in RNA interference, monitoring disease progression in animals, and breakthroughs in chemistry and genome engineering indicates invaluable opportunities for improving livestock health standards, including inhibition and control of animal disease, which should be used.

## Conclusion

In conclusion, the evaluation of the efficiency of the prepared lentivirus-mediated interfering RNAs, employing real-time RT-PCR and assaying the infectious BDV titer, revealed that an impressive success in the inhibition of virus replication was achieved even though shRNA 2 and 3 showed a more proper function than the others.

## Data Availability Statement

The original contributions presented in the study are included in the article/supplementary material, further inquiries can be directed to the corresponding author/s.

## Author Contributions

MA, LA, BS, and AM performed the experiments. AM, BS, and LA wrote the paper. All the authors conceived, designed the experiments, read, and approved the final manuscript.

## Conflict of Interest

The authors declare that the research was conducted in the absence of any commercial or financial relationships that could be construed as a potential conflict of interest.

## Publisher's Note

All claims expressed in this article are solely those of the authors and do not necessarily represent those of their affiliated organizations, or those of the publisher, the editors and the reviewers. Any product that may be evaluated in this article, or claim that may be made by its manufacturer, is not guaranteed or endorsed by the publisher.
